# Dual Role of Respiratory Syncytial Virus Glycoprotein Fragment as a Mucosal Immunogen and Chemotactic Adjuvant

**DOI:** 10.1371/journal.pone.0032226

**Published:** 2012-02-27

**Authors:** Sol Kim, Dong-Hyun Joo, Jee-Boong Lee, Byoung-Shik Shim, In Su Cheon, Ji-Eun Jang, Ho-Hyun Song, Kyung-Hyo Kim, Man Ki Song, Jun Chang

**Affiliations:** 1 Division of Life & Pharmaceutical Sciences, Ewha Womans University, Seoul, Korea; 2 Laboratory Science Division, International Vaccine Institute, Seoul, Korea; 3 Department of Pediatrics, Ewha Womans University School of Medicine, Seoul, Korea; Veterinary Laboratories Agency, United Kingdom

## Abstract

Respiratory syncytial virus (RSV) is a major cause of severe lower respiratory tract disease in infancy and early childhood. Despite its importance as a pathogen, there is no licensed vaccine to prevent RSV infection. The G glycoprotein of RSV, a major attachment protein, is a potentially important target for protective antiviral immune responses and has been shown to exhibit chemotactic activity through CX3C mimicry. Here, we show that sublingual or intranasal immunization of a purified G protein fragment of amino acids from 131 to 230, designated Gcf, induces strong serum IgG and mucosal IgA responses. Interestingly, these antibody responses could be elicited by Gcf even in the absence of any adjuvant, indicating a novel self-adjuvanting property of our vaccine candidate. Gcf exhibited potent chemotactic activity in *in vitro* cell migration assay and cysteine residues are necessary for chemotactic activity and self-adjuvanticity of Gcf *in vivo*. Mucosal immunization with Gcf also provides protection against RSV challenge without any significant lung eosinophilia or vaccine-induced weight loss. Together, our data demonstrate that mucosal administration of Gcf vaccine elicits beneficial protective immunity and represents a promising vaccine regimen preventing RSV infection.

## Introduction

Respiratory syncytial virus (RSV) is the leading cause of serious respiratory tract disease in infants and young children worldwide. RSV is also an important viral pathogen of lower respiratory tract illness in immunocompromised patients and the elderly [1], [2], [3]. Despite the importance of RSV as a respiratory pathogen, there is no licensed vaccine currently available. Thus, development of an effective and safe RSV vaccine is urgently required.

The RSV glycoprotein (G) was identified as the major RSV attachment protein [4], and is thought to be important for protection against RSV infection [5]. The RSV G protein contains a CX3C motif at amino acids 182 to 186 in the central conserved region and binds to CX3CR1, inducing leukocyte chemotaxis [6]. Numerous studies have suggested that immunization of RSV G is associated with the induction of polarized Th2 type responses which leads to pulmonary eosinophilia upon RSV challenge of G-immunized mice [7], [8], [9], [10], [11]. However, we have recently shown that single intranasal immunization of recombinant adenovirus expressing a fragment of RSV G protein induces strong antibody responses without atypical pulmonary inflammation after RSV challenge [12], demonstrating that G protein could provide protection without enhanced lung pathology, depending on the vehicle and/or method of delivery.

Mucosal vaccination against pathogens generally offers several attractive advantages to conventional systemic vaccination, such as higher levels of antibodies and protection at the sites of pathogen entry, and non-invasive and convenient administration. Since mucosal vaccination targets specific mucosal area and mostly induces protective immunity at the site of administration, intranasal immunization is thought to be most appropriate for vaccines against respiratory pathogens. However, a safety issue caused by the redirection of the vaccine to the central nerve system has remained about intranasal administration [13], [14]. In contrast, the sublingual route has been used for allergen immunotherapy and is generally known to induce T-cell anergy, immune deviation, and activation of regulatory T cells [15], [16]. However, it has recently been shown that sublingual administration of a protein antigen with cholera toxin (CT) adjuvant can induce strong antigen-specific antibody and CTL responses that are comparable to those induced by intranasal administration [17].

In the present study, we targeted the RSV G protein fragment of residues 131 to 230 as a vaccine candidate, and expressed and purified this fragment from *E. coli*. We show that sublingual or intranasal immunization of G protein fragment induces strong serum IgG responses as well as mucosal IgA responses, and provides potent protection against RSV challenge, even in the absence of any adjuvant.

## Results

### Construction and purification of RSV G protein fragment, Gcf

**Figure 1:**
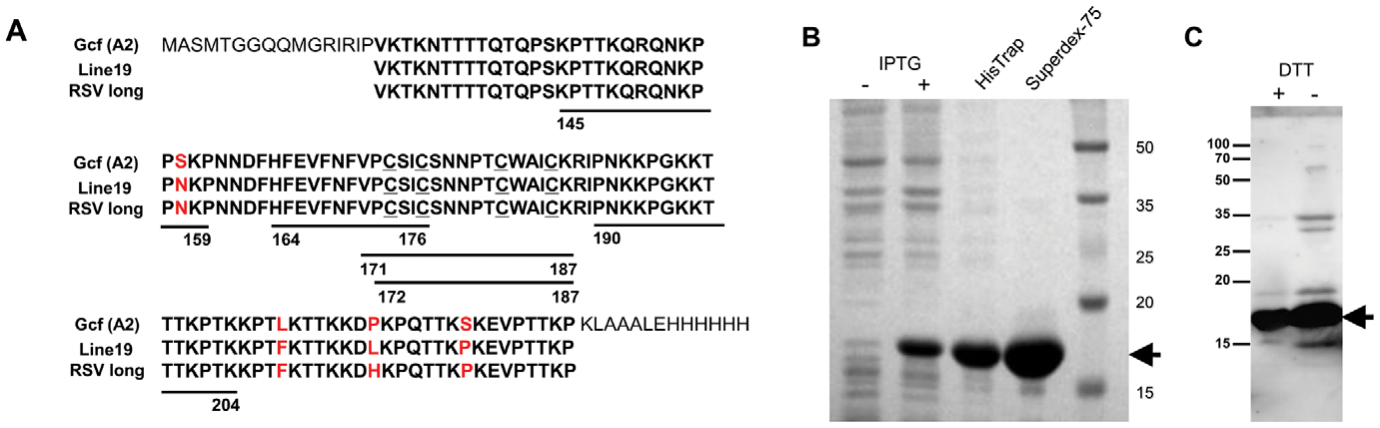
Expression and purification of recombinant G protein fragment, Gcf. (A) Deduced amino-acid sequence of the recombinant RSV G protein fragment (Gcf). RSV G-derived amino acids are indicated in bold letters and the conserved four cysteine residues are underlined. For comparison, the same regions of RSV long and line 19 strains are aligned. The five regions of B-cell epitopes defined by the previous studies are indicated by residue numbers and thick lines below the aligned sequences. The expression and purification steps of the Gcf by affinity (HisTrap) and gel filtration chromatography (Superdex-75) was confirmed by SDS-PAGE (B) and western blotting (C) with anti-RSV polyclonal antibody under reducing condition (+DTT) or non-reducing condition (−DTT).

The human RSV G glycoprotein contains a central conserved region that includes four completely conserved cysteine residues. Antigenic analyses of G protein with monoclonal antibodies suggest the importance of this region as both immunogen and an antigen [18], [19]. Based on the G protein architecture, we designed a subunit vaccine that encompasses the neutralizing epitopes of the central conserved region and the important structural elements. This vaccine construct included amino acid sequences from 131 to 230 and was cloned into pET-21d expression vector; the resulting protein was designated as Gcf (Fig. 1A). The Gcf protein was purified from *E. coli* and analyzed by SDS-PAGE and western blotting, showing a predominant band at the expected molecular weight of ∼17 kDa, corresponding to monomeric form (Fig. 1B and C).

### Humoral immune response to RSV G protein fragment

**Figure 2:**
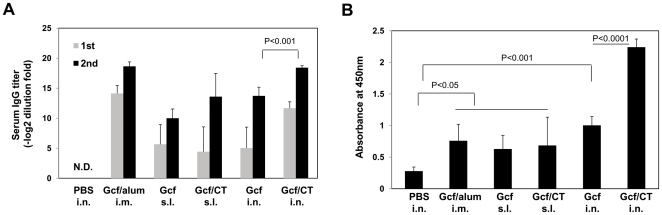
Characterization of humoral responses induced by Gcf immunization. (A) BALB/c mice were immunized twice on day 0 and day 14 with 20 µg of recombinant Gcf via indicated routes and serum IgG antibody titers specific for Gcf were measured by ELISA two weeks after the first and second immunizations. The results represent Log_2_ endpoint values from individual mice (*n* = 6). The results are a representative of three independent experiments. (B) Absorbances for RSV-specific IgA were measured in the BAL fluid three weeks after the second immunization. N.D., not detected.

We next examined whether purified Gcf could elicit Ag-specific immune responses *in vivo*. Recently, sublingual immunization was shown to efficiently induce both mucosal and systemic antibody responses [17]. To compare the immune responses induced by different routes of vaccination, groups of BALB/c mice were inoculated via sublingual (s.l.), intranasal (i.n.), or intramuscular (i.m.) route with 20 µg of Gcf with or without adjuvant on days 0 and 14. For comparison, live RSV A2 virus was given by i.n. instillation or formalin-inactivated RSV particle (FI-RSV) was injected into the foot-pad, if necessary. Two weeks after each immunization, animals were bled and the sera of individual animals were examined for G-specific IgG by ELISA. Serum IgG antibody responses were readily detected in all groups of immune mice (Fig. 2A). Intramuscular injection with alum and intranasal immunization elicited slightly higher serum IgG responses than sublingual immunization. Intriguingly, i.n. or s.l. immunization of Gcf alone induced significant serum IgG responses,and the antibody response induced following vaccination by the i.n. route was further enhanced when Gcf was given together with CT adjuvant.

Secretory antibodies are the first line of host defense against aerial pathogens and are known to be important correlates of protection against RSV [20]. To examine whether Gcf vaccination also elicits mucosal antibody response in the respiratory tract, bronchoalveolar lavage (BAL) was performed three weeks after the second immunization and levels of specific IgA in the BAL were determined by ELISA. As shown in Fig. 2B, the levels of mucosal IgA were elevated in all immune groups, and the Gcf/CT i.n. group showed the highest levels of mucosal IgA. These results demonstrate that mucosal immunization of purified Gcf with or without adjuvant successfully induces secretory IgA responses.

### T-cell responses to G protein fragment and no enhanced diseases

**Figure 3:**
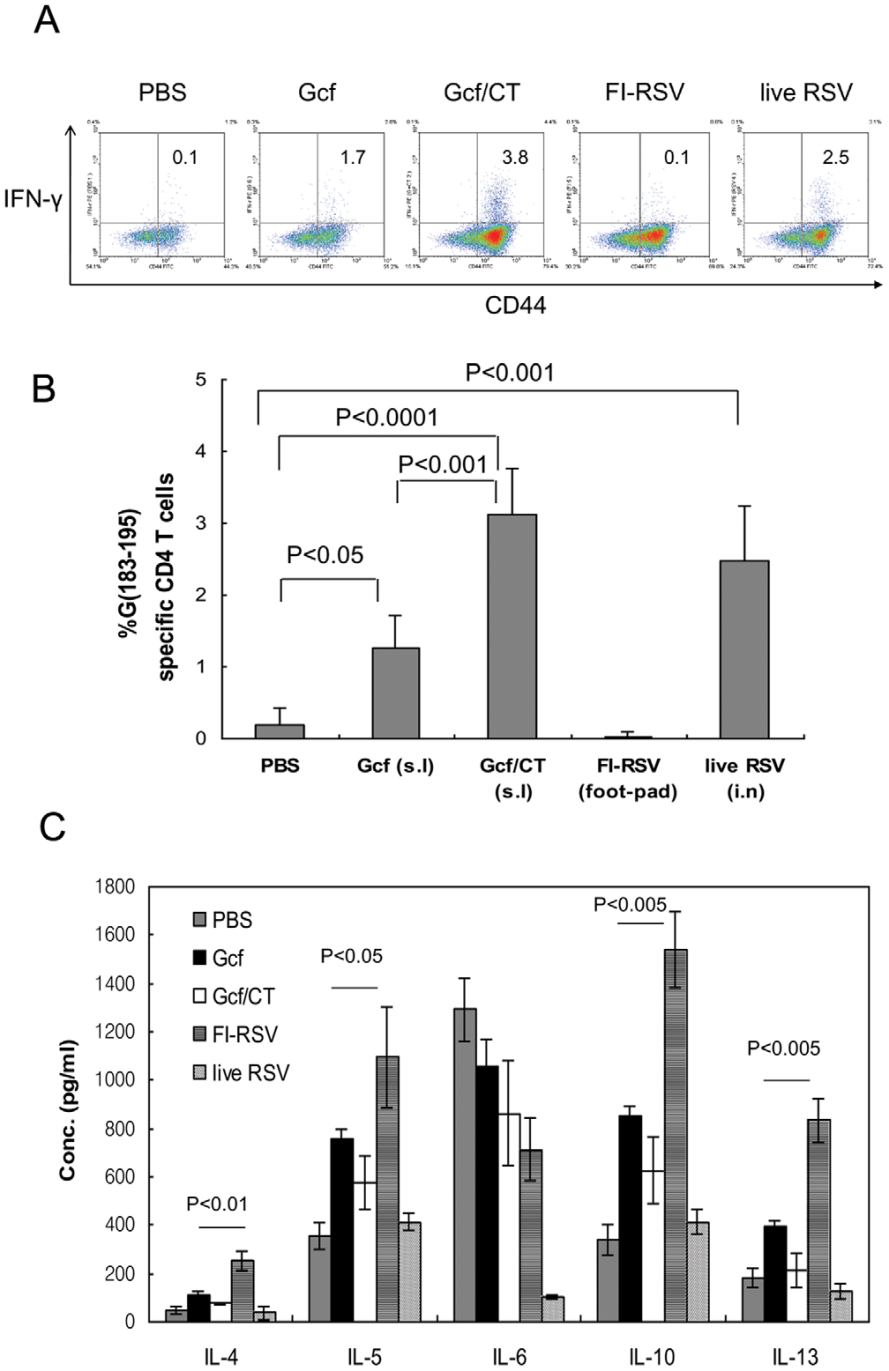
G-specific CD4 T-cell response in Gcf-immune mice. (A) Mice were immunized twice with Gcf s.l., once with FI-RSV by food-pad injection, or 1×10^5^ PFU of live RSV A2 via i.n. route, and then challenged with RSV three weeks after final immunization. Lung mononuclear cells were prepared from the lungs of the same group of mice (*n* = 6) four days after challenge and then stimulated with G(183–195) peptide in the presence of Brefeldin A. Cells were stained for CD4, CD44, and IFN-γ, and analyzed by flow cytometry. Cells gated for CD4 expression are shown in each dot plot and the percentages represent the frequency of G-specific IFN-γ-positive cells The numbers in the upper right quadrant indicate the percentage of IFN-γ+ cells among total lung CD4+ cells after subtracting the percentages by isotype control staining of the same samples. (B) Average data represent mean ± SD (*n* = 6). The results are a representative of two independent experiments. (C) Cytokine levels in the lung supernatant were assessed at day 5 post-challenge by multiplex antibody-based assay (FlowCytomix).

Priming of mice with FI-RSV or vaccinia virus expressing G protein induces Th2-biased responses and enhances diseases characterized by pulmonary eosinophilia following RSV challenge in a mouse model [21], [22]. It is thought that G-induced enhanced disease is associated with dominant G-specific Th responses in the absence of CTL response [11], [21]. Since Gcf contains I-E^d^-restricted Th epitope, we examined whether Gcf immunization primes G-specific CD4 T cells and subsequently induces enhanced disease. To this end, Gcf-, FI-RSV or live RSV-immune mice were challenged with RSV, and checked for G-specific CD4 T cells by intracellular cytokine staining with CD44 as an activated T-cell marker. As shown in Fig. 3, the G-specific IFN-γ-producing CD4 T-cell response was low in the lungs of s.l. Gcf-immunized group (≤1.4% of gated CD4 T cells), while slightly stronger responses were observed in the Gcf/CT or live RSV immunized group (∼3.0% and ∼2.5% of gated CD4 T cells on average, respectively; Fig. 3A and B).

It is true that Th2-biased response by G protein priming is one of important issues of RSV vaccine development. Thus, we performed an experiment to check the level of Th2 type cytokines after live RSV challenge of immunized mice. IL-4, IL-5, IL-6, IL-10, or IL-13 levels in lung supernatants at day 5 post-challenge were measured by multiplex antibody-based assay as described in the Materials and Methods. The levels of IL-4, IL-5, IL-10, or IL-13 detected in lung samples from mice immunized with FI-RSV were statistically greater than those of mice immunized with Gcf or Gcf/CT (p<0.05∼0.005, Fig. 3C). The similarly low levels of type 2 cytokines were observed in Gcf or Gcf/CT immunized mice.

**Figure 4:**
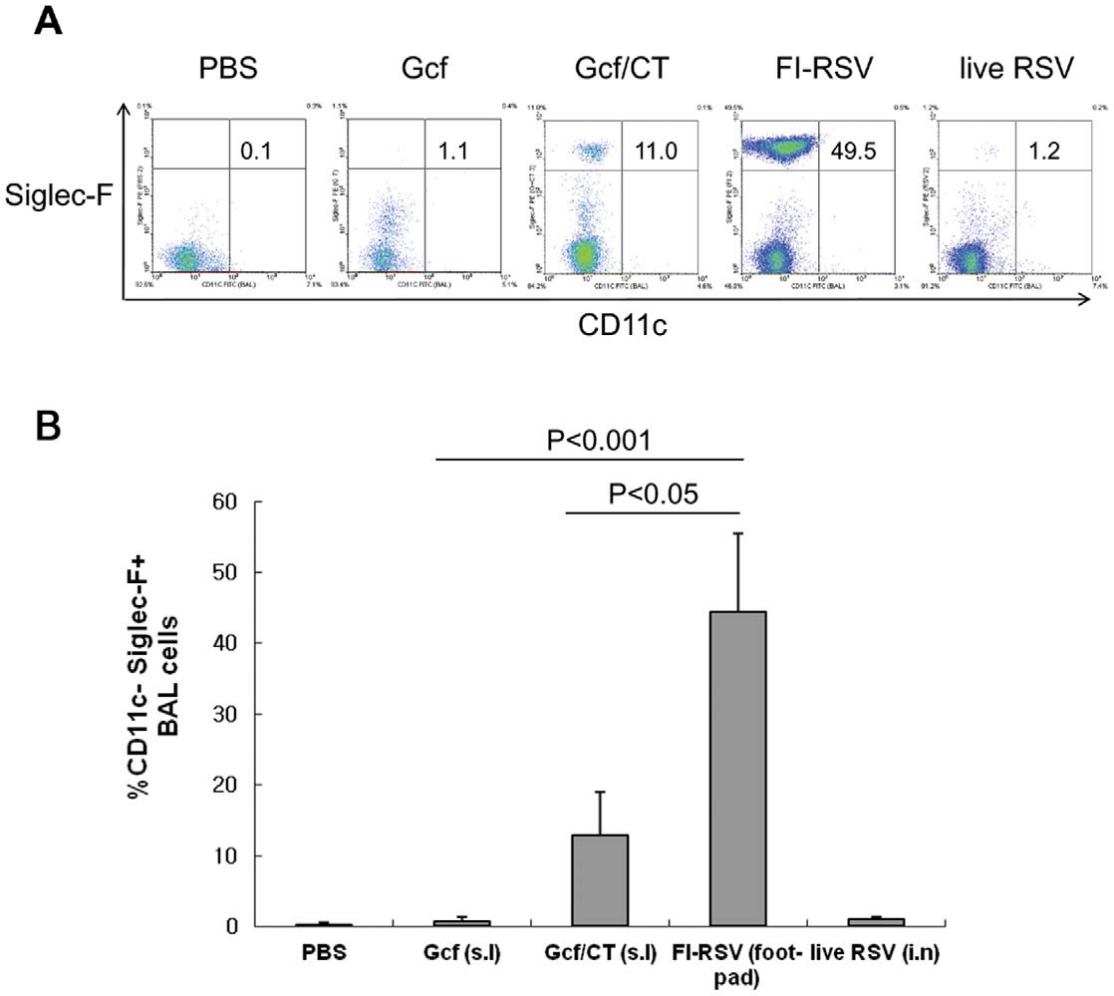
Low lung eosinophilia in Gcf-immune mice. (A) Mice were immunized and challenged as in Fig. 3. BAL was performed five days after RSV challenge, and cell pellets were surface stained with anti-CD45, Siglec-F, and CD11c and eosinophils were quantitated among CD45-positive-gated cells. The numbers in the upper right quadrant indicate the percentage of Siglec-F+CD11c- cells among total CD45+ cells. (B) Average data represent mean ± SD (*n* = 6). The results are a representative of three independent experiments.

To determine whether the mucosal immunization of Gcf potentiates eosinophilia, the levels of eosinophilia in BAL of the immune mice were examined by flow cytometry five days after challenge using antibodies directed to Siglec-F, CD45, and CD11c as described previously [23]. A weak infiltrate of eosinophils, less than 2% of the total CD45+ BAL cells, was observed in Gcf-immune mice while more pronounced eosinophil influx was detected in the Gcf/CT group (∼13.1% on average, Fig. 4A and B). The level of eosinophil influx in Gcf-immune mice was similar in magnitude to that observed in the control mice or mice previously injected with live RSV virus. As a positive control, eosinophilia was markedly enhanced in FI-RSV-immune animals (Fig. 4; ∼45% of total CD45+ BAL cells). These results suggest that mucosal Gcf immunization in the absence of adjuvant barely increased the risk of development of vaccine-induced eosinophilia.

### Protective efficacy of mucosal Gcf vaccine against RSV challenge

**Figure 5:**
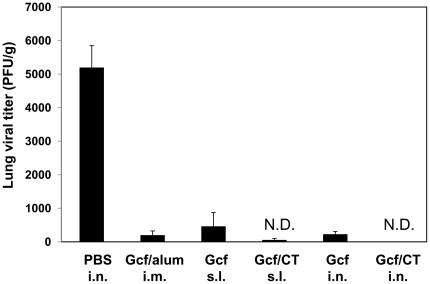
Protective efficacy of mucosal vaccination with Gcf. Each group of immune mice was challenged with 1×10^6^ PFU RSV A2 at 4 weeks after immunization and the levels of RSV replication in the lungs were determined by plaque assay at day 4 post challenge. Results are expressed as the mean ± SEM (*n* = 6). The limit of detection was 200 PFU/gram of lungs. N.D., not detected.

Our results demonstrate that mucosal vaccination of Gcf protein successfully induces humoral responses. To test whether the immunity induced by mucosal Gcf vaccination confers protection against virus challenge, the immune mice were challenged with live RSV A2 virus at four weeks after immunization. While there was active RSV replication in the lungs of the control mice, s.l. or i.n. immunization of Gcf/CT adjuvant prevented any detectable RSV replication in the lungs at the peak of viral replication (Fig. 5). A group of mice previously injected with FI-RSV or live RSV A2 virus also exhibited complete protection from the challenge (data not shown). It is also noteworthy that i.n. or s.l. immunization of Gcf alone resulted in almost complete protection (Fig. 5). In keeping with potent lung protection, there was no significant weight loss upon RSV challenge in Gcf-immune mice, while FI-RSV-scarified mice showed significant weight loss and disease scores (data not shown). Together, these results suggest that mucosal Gcf vaccination gives rise to protective immunity in the absence of priming of pathologic CD4 T cells and subsequent vaccine-enhanced diseases.

### Chemotactic activity of recombinant Gcf

**Figure 6:**
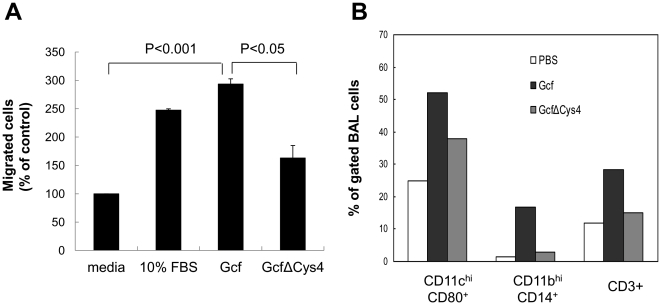
Chemotactic activity mediated by Gcf. (A) *In vitro* chemotaxis was analyzed using transwell insert plate with 5 µm pore size. 10 µg of Gcf or GcfΔCys4 were added to the bottom chamber and 5×10^5^ THP-1 cells were added to the top chamber of the plate. Media alone or media with 10% FBS was added as negative or positive control, respectively. The assembled plates were incubated at 37°C for 5 h. Cells migrated to the bottom chamber were collected and counted at least 3 times. Data are expressed as mean ± SEM percentages of migrated cells over negative control (media alone) from at least three independent experiments. (B) *In vivo* chemotaxis was examined by intranasal administration of Gcf or GcfΔCys4 in the absence of adjuvant and flow cytometric analysis of BAL cells collected 48 h after injection. BAL cells were pooled from at least three mice for each group and the percentages of cells were calculated after gating viable cells on FSC/SSC.

It is interesting to note that mucosal immunization of Gcf without any adjuvant also elicited strong humoral responses. The core domain of G protein contains a CX3C motif that has limited homology with the CX3C domain of the chemokine fractalkine [6]. Since Gcf contains this CX3C motif, we examined whether the purified Gcf exhibits chemotactic activity in an *in vitro* chemotaxis assay. As a control, a mutant Gcf, GcfΔCys4, was generated in which four cysteine residues were substituted with alanine. When THP-1 cells were incubated with wild type Gcf and 10% FBS as a positive control [24], the numbers of migrating cells increased ∼3.5-fold and ∼5-fold, respectively (Fig. 6A). However, the mutant GcfΔCys4 exhibited significantly decreased chemotactic activity compared to wild type Gcf, indicating that cysteine residues are necessary for Gcf-mediated chemotactic activity.

Next, we determined whether *in vivo* administration of Gcf without adjuvant recruits immune cells to the site of injection. As shown in Fig. 6B, intranasal administration of Gcf significantly increased infiltration of CD11c^hi^CD80^+^ (possibly dendritic cells), CD11b^hi^CD14^+^ (macrophages), and CD3^+^ (T cells and NKT cells) cells to the lungs, while GcfΔCys4 did not.

**Figure 7:**
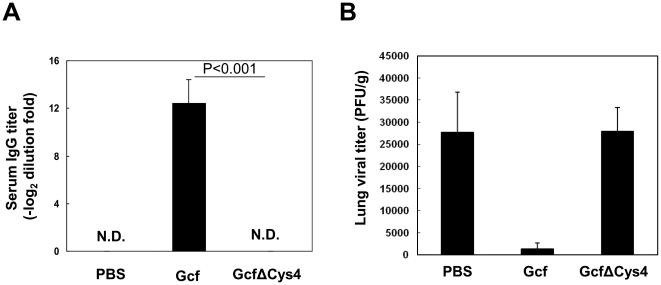
Cysteine residues of Gcf are necessary for the induction of specific antibody response and protection. (A) BALB/c mice were immunized twice with 20 µg of recombinant Gcf or mutant GcfΔCys4 via intranasal route and specific serum antibody titers were measured by ELISA two weeks after immunization. The results represent Log_2_ endpoint values from at least five individual mice. (B) Each group of i.n. immunized mice was challenged with 2×10^6^ PFU RSV A2 at 4 weeks after boost immunization and the levels of RSV replication in the lungs were determined by plaque assay at day 5 post challenge. Results are expressed as the mean ± SEM (*n* = 6). The limit of detection was 200 PFU/gram of lungs.

To determine whether the chemotactic activity of recombinant Gcf is indeed necessary for the induction of specific immune responses without any adjuvant, mice were i.n. immunized with wild type Gcf or mutant GcfΔCys4 alone and antibody responses were checked. As shown in Fig. 7A, specific serum antibody response was not detected above background in the mutant GcfΔCys4-immune group, suggesting that cysteine residues and chemotactic activity of Gcf are required for its immunogenicity in the absence of adjuvant. To determine the protective efficacy of GcfΔCys4, we examined the lung of immune mice for viral replication after challenge with live RSV. Four weeks after the boost immunization, mice were challenged intranasally with live RSV. As shown in Fig. 7B, there was active RSV replication in the lung of the PBS immunized mice and GcfΔCys4 immunized mice. Our results indicate that protective immunity of Gcf requires the conserved cysteine residues.

## Discussion

RSV vaccine has been sought since the virus was discovered in the 1950s. Due to its tremendous disease burden and the limited availability of possible prophylactic methods, the need for a safe and effective RSV vaccine is greater than ever. However, many hurdles have hampered the development of RSV vaccine: (i) The higher standards for safety are applied due to possible vaccine-enhanced immunopathology and the relatively immature state of young vacinees, (ii) high prevalence of maternal antibodies might diminish the efficacy of some vaccine candidates like live-attenuated virus, and (iii) frequent reinfection with the same virus might be related to short term memory and possible immunoregulatory mechanisms exerted by RSV. Though various strategies have been employed to develop RSV vaccine, our aim in the current study is to develop mucosal RSV vaccine candidates that are safe and effective. In addition to the superior ability of mucosal vaccination to induce local mucosal immunity compared to systemic vaccination, mucosal vaccination also offers many additional advantages such as a needle-free, non-invasive application and convenient delivery without special training. Thus, we adopted mucosal administration of our vaccine candidate through the intranasal or sublingual route, which efficiently elicited respiratory tract immunity.

Our study indicates that sublingual immunization and intranasal immunization of RSV G protein fragment effectively induce both mucosal and systemic antibody immunity. Also, administration of recombinant Gcf protein in the absence of any adjuvant is sufficient to induce humoral responses that provide partial but potent protection against live virus challenge. Due to a certain degree of mucosal compartmentalization, the intranasal route for the delivery of vaccines against respiratory pathogens is thought to be the most effective for induction of protective mucosal immunity. However, there has been a safety concern in intranasal administration due to retrograde passage of delivered antigen/adjuvant materials through the olfactory epithelium [25], [26]. In contrast to the intranasal route, sublingual administration did not result in such retrograde passage of vaccines to the central nervous system [17]. In this regard, sublingual immunization of our Gcf vaccine might be a safer choice for delivery of RSV vaccine and our findings provide a rationale for further development of sublingual Gcf vaccination. More importantly, Gcf vaccination induced this protective immunity without use of adjuvant. Normally, protein antigens without adjuvant administered via the mucosal route are reported to be only weakly immunogenic or tolerogenic [27], and thus require a mucosal adjuvant such as cholera toxin (CT) to induce antigen-specific response. Thus, vaccination of Gcf without adjuvant will be another safety advantage in the future development, because most adjuvants used in vaccines cause unwanted side effects in human.

We targeted the most conserved region of the G protein which contains several neutralizing epitopes and an important structural motif. Within the central conserved domain, 13 residues (amino acids 164 to 176) and four cysteine residues (Cys-173, Cys-176, Cys-182, Cys-186) are completely conserved among HRSV-A strains. The secondary structure of this central domain is thought to be a tight turn, and disulfide bridges could occur between Cys-173 and Cys-186, and between Cys-176 and Cys-183 [28]. Five protective B-cell epitopes were identified within this conserved region of G protein, that were recognized by human sera as well as by murine mAb [29]. Previously, vaccination of bacterially expressed protein vaccine, BBG2Na, which contained residues 130 to 230 of RSV A2 G protein successfully induced protective immunity against homologous virus challenge [30]. This protein vaccine also induced cross-protective immunity against RSV B infection, although the duration of protection was relatively shorter than that for RSV A virus [31]. In contrast, subgroup-specific native G proteins did not induce significant cross-protective immunity [32]. A possible explanation for the cross-protective immunity induced by the bacterially expressed vaccine is that the absence of glycosylation increases the immunogenicity of one or more of the five protective epitopes, which are mostly conserved between two subgroups. Thus, vaccination of bacterially expressed Gcf may provide broader than expected cross-protection against a wide range of RSV strains. This possibility is under investigation using clinical isolates belonging to the B subgroup.

Our study clearly demonstrated that Gcf exhibits chemotactic activity both *in vitro* and *in vivo*. The phenotypes of cells recruited by Gcf administration were CD11c^hi^, CD11b^hi^, and CD3^+^ cells, which are all known to express CX3CR1 [33], [34], [35]. Thus, it is likely that self-adjuvanting effect of Gcf is associated with active recruitment of antigen-presenting immune cells through CX3C-CX3CR1 interaction. Several features of G protein have marked similarities to the CX3C chemokine, fractalkine: the conserved CX3C motif region of G protein has high homology with the chemokine domain of fractalkine (∼40%), both G protein and fractalkine exist as membrane-bound and secreted forms, and both proteins have heparin-binding domains that bind to glycosaminoglycans on the cell surface [36], [37]. Indeed, it has been previously shown that G protein interacts with CX3CR1 in a manner similar to fractalkine, suggesting that G protein may modulate the immune response [6]. While the central CX3C region seems to be implicated in disease pathogenesis, the same region may also contribute to the induction of protective immunity by blocking G protein-CX3CR1 interaction during the course of RSV infection [38]. In our study, the humoral immunity raised by Gcf vaccination conferred protection against RSV infection, supporting the protective role of antibodies to this region of G protein. Interestingly, the previous studies have shown that intranasal immunization of G128–229 elicited poorly immunogenic and partially protective responses without adjuvant [39]. Our results, however, differ from this study on several aspects: mucosal immunization of our Gcf vaccine expressing soluble form of G131–230 elicited serum IgG and RSV-specific CD4 T-cell response, which led to protective immunity in BALB/c mice without adjuvant. We think that poorly immunogenic responses of G128–229 immunization might be due to fusion with bacterial thioredoxin, which might affect the conformation of G fragment. The similar region of G (130–230) had previously been reported in bacteria as a fusion with the albumin-binding region of streptococcal protein G [30]. It also induced poorly humoral immune responses without adjuvant. Relatively strong immunogenicity of Gcf may be due to expression of soluble Gcf by itself, and soluble Gcf configuration without any fusion partner may be important for effective self-adjuvanticity by chemotaxis and subsequent activation of immune cells. However, we cannot exclude the possibility that the several experimental conditions like injection volume, the amount of administered antigen, and the buffer composition are different from each other and these make it difficult to interpret and directly compare those results and ours. Based on our results, we believe that native Gcf conformation without carrier protein is more effective in inducing the immune responses in the absence of adjuvant. Further study might be necessary to elucidate the mechanisms of this difference.

In conclusion, we demonstrate that procaryotically expressed G protein fragment, Gcf, is a promising candidate for mucosal RSV vaccine and potent protective immunity could be induced by sublingual or intranasal administration even in the absence of adjuvant. Our study also clearly demonstrated that the cysteine residues and chemotactic activity are necessary for the induction of specific immunity by Gcf in the absence of adjuvant, proposing a novel self-adjuvanticity of Gcf. Further study is necessary to address the exact mechanisms involved in this newly-defined property of Gcf mucosal vaccine and to develop Gcf as a mucosal vaccine for human use.

## Materials and Methods

### Ethics statement

All animal experiments were also approved by Ewha Womans University's Institutional Animal Care and Use Committee (Approval ID: 2010-9-4).

### Preparation of RSV stock

RSV A2 strain was propagated in HEp-2 cells (ATCC, Manassas, VA) in DMEM (Life Technologies, Gaithersburg, MD) supplemented with 3% heat-inactivated FCS, 2 mM glutamine, 20 mM HEPES, nonessential amino acids, penicillin, and streptomycin. Virus was harvested at day 4∼5 after infection when the infected HEp-2 cells exhibited maximal cytopathic effect. In brief, cells were disrupted by three cycles of freeze-thawing, then sonicated for 1 min, and centrifuged at 300 g for 10 min. The supernatants were collected, combined with cleared supernatants from the infected HEp-2 culture, and centrifuged at 75,000 g for 1 hour. The pellets were resuspended with serum-free MEM by using 25-gauge needle and brief sonication, and the final titer was determined by standard plaque assay.

### Construction of expression vectors and purification of RSV G protein fragment

The coding sequence of RSV G protein spanning from amino acid residues 131 to 230 (RSV A2 strain) was amplified from cDNA by PCR and cloned into the EcoR I and Hind III sites of pET-21d(+) vector (Novagen). A mutant DNA in which four cysteine residues (Cys-172, Cys-176, Cys-182, Cys-186) were substituted with alanine was generated by mega-PCR method with mutagenic primers [40]. The constructed plasmid was transformed into *E. coli BL21(DE3)* (Novagen) and overexpression was induced by adding Isopropyl β-_D_-1-thiogalactopyranoside (IPTG, Takara, Shiga, Japan). After centrifugation, bacterial cells were suspended in binding buffer (20 mM Tris, 0.5 M NaCl, pH 8.0) and sonicated. Soluble and insoluble proteins were separated by centrifugation at 40,000×g for 20 min and clear supernatants were used for further purification. Expressed G protein fragment (Gcf) and mutant Gcf (GcfΔCys4) were purified by affinity chromatography using HisTrap column (GE Healthcare). After washing with binding buffer containing 20 mM imidazole, loaded proteins were eluted with elution buffer (500 mM imidazole, 20 mM Tris, 0.5 M NaCl, pH 7.4). Eluted protein fractions were further purified using Superdex-75 column after equilibration with PBS (GE Healthcare). The purified protein was treated with 1% Triton X-114 to remove endotoxins for 30 min at 4°C, followed by incubation at 37°C for 20 min. The phase containing endotoxin was separated by centrifugation. This cycle was repeated five times. Then, the protein was incubated with SM-2 beads (Bio-Rad, Hercules, CA) for 2 h at 4°C to remove residual Triton X-114 and filtered through spin-X column (Costar, Washington, DC). The endotoxin levels in the protein preparation were measured by the limulus amebocyte lysate (LAL) assay kit according to the manufacturer's instructions (Lonza, Switzerland). The endotoxin level was less than 5 EU/mg. Protein concentrations were measured by Bio-Rad Protein assay (Bio-Rad Laboratories). The centrifugal filter, Amicon® ultra (Millipore, Bedford, MA) was used for additional concentration. To visualize the purification, samples were resolved on 15% SDS-PAGE and stained with Coomassie Brilliant Blue. The purity of Gcf vaccine was also verified by western blotting using RSV-specific goat polyclonal antibody (US Biological) and HRP-conjugated anti-goat Ig antibody (Zymed Laboratories, San Francisco, CA). Purified proteins were stored at −80°C in aliquots until use.

### Mice immunization and challenge

Female BALB/c mice were purchased from Charles River Laboratories Inc. (Yokohama, Japan) and kept under specific pathogen-free conditions. For the immunization, 6 to 8-week-old mice were inoculated twice on day 0 and day 14 with 20 µg of purified G protein fragment via the sublingual (s.l.) or intranasal (i.n.) route. For s.l. immunization, mice were anesthetized by i.p. injection of ketamine and recombinant RSV G fragment with or without cholera toxin (2 µg) in 15 µl were delivered underneath the tongue. For i.n. immunization, mice were lightly anesthetized by isoflurane inhalation, and 50 µl of vaccine or PBS were applied to the left nostril. FI-RSV (1×10^5^ PFU in 50 µl) with aluminum hydroxide (Sigma-Aldrich, Seoul, Korea) was administered once through the foot pad of anesthetized mice. As a positive control, live RSV (1×10^5^ PFU) was i.n. delivered once. Three to four weeks after the last immunization, the mice were challenged i.n. with 1×10^6^ or 2×10^6^ PFU of live RSV A2, if necessary. All animal studies were performed with approval of our Institutional Animal Care and Use Committee (Approval No. 2010-9-4).

### ELISA

Blood was obtained from the retro-orbital plexus via a heparinized capillary tube, collected in an eppendorf tube and centrifuged; serum was obtained and stored at −20°C. Antibody titers from immunized mice specific for RSV G protein were measured by direct ELISA. Briefly, 96-well plates were coated overnight with 100 µl/well of 2×10^3^ PFU of purified RSV A2 virus diluted in PBS, and blocked with PBS containing 2% BSA and 0.05% Tween 20 for 2 h. Sera or BAL fluids were then added in serial dilutions and incubated for 2 h. The plates were washed five times with PBS containing 0.05% Tween 20 and incubated for 1 h with varying dilutions of HRP-conjugated affinity-purified rabbit anti-mouse total IgG or IgA secondary antibody (Zymed Laboratories, San Francisco, CA). The plates were washed three times, developed with 3,3′,5,5′-tetramethylbenzidine, stopped with 1M H_3_PO_4_, and analyzed at 450 nm by a Thermo ELISA plate reader. Naïve pooled sera from age-matched mice were used as a negative reference sera and if OD differences of the samples to the negative reference at the same dilution were not greater than 0.1, these values were regarded as ‘not detected’. Cut-off points were calculated by subtracting the background values of the negative reference from those of experimental samples at the same dilutions and subsequent linear regression analysis.

### RSV titer in the lung

Four days after RSV challenge, subsets of mice were euthanized and the lungs were removed into Eagle's modified essential medium. The tissues were then processed through a steel screen to obtain single-cell suspensions, and particulate matter was removed by passing through 70-µm cell strainer (BD Labware, Franklin Lakes, NJ). The supernatants were collected and RSV titers in the supernatants were measured by plaque assay on subconfluent HEp-2 monolayers. The data are expressed as PFU per gram of lung tissue.

### Bronchoalveolar lavage (BAL)

Five days post challenge, a subset of mice from each group was sacrificed and tracheotomy was performed. The lung airways were washed with 0.8 ml of PBS containing 1% FBS. The collected BAL cells and supernatants were used in counting leukocytes by flow cytometry and measuring secretory IgA titers, respectively.

### Preparation of lung lymphocytes and flow cytometric analysis

The lungs were perfused with 5 ml of PBS containing 10 U/ml heparin, and then removed and processed through a steel screen to obtain single-cell suspensions; particulate matter was removed by passage through 70-µm Falcon cell strainer (BD Labware). Freshly explanted BAL or lung cells were purified by density gradient centrifugation and were stained in a buffer (PBS/3% FBS/0.09% NaN_3_) with fluorochrome-conjugated antibodies. The antibodies used were anti-CD3e (clone 145-2C11), anti-CD4 (RM4-5), anti-CD11b (M1/70), anti-CD11c (HL3), anti-CD14 (rmC5-3), anti-CD44 (IM7), anti-CD80 (16-10A1), and anti-Siglec F (E50–2440). All antibodies were purchased from BD Bioscience (San Diego, CA). After staining, cells were fixed in PBS/2% (wt/vol) paraformaldehyde, and events were acquired using a FACSCalibur® flow cytometer (BD Biosciences). To enumerate the cytokine-producing cells, cells were stimulated with 10 µM G(183–195) peptide (WAICKRIPNKKPG), and incubated for 5 h in the presence of Brefeldin A (5 µg/ml; Sigma-Aldrich). Then, cells were stained for surface markers, washed, fixed and permeabilized with FACS buffer containing 0.5% saponin (Sigma-Aldrich), and stained for IFN-γ. The antibodies used were anti-IFN-γ (clone XMG1.2) or its control isotype antibody (rat IgG1). Dead cells were excluded on the basis of forward and side light scatter patterns. Data were collected using CELLQuest® software (BD Biosciences) and analyzed with CELLQuest® and WinMDI version 2.9 software (The Scripps Research Institute, La Jolla, CA). Lung supernatants were collected for analysis with the FlowCytomix (eBioscience), according to the protocol. Kits containing antibody beads (IL-4, IL-5, IL-6, IL-10, IL-13) were used to measure cytokine production in each of the samples.

### Chemotaxis assay

Chemotaxis was analyzed using 24-well tissue-culture-treated transwell insert plate with 5 µm pore size (Costar, Corning, NY). 10 µg of Gcf or GcfΔCys4 in DMEM with 1% BSA were added to the bottom chamber and a total of 5×10^5^ THP-1 cells, human monocyte leukemia cell line, were added to the top chamber of the plate. The assembled plates were incubated at 37°C for 5 h. Cells migrated to the bottom chamber were collected and counted in a blinded manner. Experiments were performed in triplicate and counts represent an average of three replicates. The experiment was repeated three times and the data were averaged.

### Data analysis

The differences were compared by an unpaired, two-tailed Student's *t*-test. The difference was considered statistically significant when *p* value ≤0.05.
